# Paranoid Thinking and Wellbeing. The Role of Doubt in Pharmacological and Metacognitive Therapies

**DOI:** 10.3389/fpsyg.2019.02099

**Published:** 2019-09-12

**Authors:** Leonor Asensio-Aguerri, Luis Beato-Fernández, Maria Stavraki, Teresa Rodríguez-Cano, Miriam Bajo, Darío Díaz

**Affiliations:** ^1^Mental Health Unit, Hospital General Universitario de Ciudad Real, Ciudad Real, Spain; ^2^Ciudad Real Medical School, Universidad de Castilla – La Mancha, Ciudad Real, Spain

**Keywords:** well-being, quality of life, paranoid thinking, doubt, Complete State Model of Health

## Abstract

**Introduction:**

Pathological confidence in one’s thoughts is a key mechanism of chronic paranoid thinking. For this reason, many of the current therapies focus on trying to reduce it. In fact, the way some antipsychotics (e.g., haloperidol) work seems to be through the induction of doubt. Because of the impact of these pathological thoughts on positive health, studying the well-being of people who experience paranoid thoughts is fundamental. The first objective of this research is to apply the Complete State Model of Health (CSMH) to a sample of patients characterized by the presence of paranoid thinking. Our second objective is to evaluate the impact of therapies based on reducing pathological confidence on patients’ well-being.

**Methods:**

Sixty participants with SCID-5 confirmed DSM-5 diagnosis related with paranoid thinking and without mood symptoms were recruited. In order to test the existence of a two continua model of mental health (CSMH), we conducted a parallel analysis and an exploratory factor analysis. To test our hypothesis regarding the partially mediating role of doubt between paranoid thinking and patients’ well-being, we conducted a biased corrected bootstrapping procedure.

**Results:**

As expected, two different unipolar dimensions emerged from the measures used to assess paranoid thinking and positive health (two continua model of mental health). When patients received metacognitive and pharmacological treatment, more paranoid thinking led to more doubt in all thoughts, which in turn affected well-being. The analyses carried out confirmed the partial mediating role of doubt.

**Conclusion:**

Despite the efficacy shown by both metacognitive therapies and antipsychotics, it seems that they not only reduce pathological confidence, but can also affect other thoughts not linked to delirium. This effect of generalization of doubt in all thoughts negatively affected patients’ well-being and quality of life.

## Introduction

Paranoid delusional thinking is one of the central symptoms of psychosis. It can appear in a wide spectrum of disorders from individuals who do not have pathologies (Penn et al., 1997), to people with delusional disorder, brief psychotic disorder, schizophreniform disorder, schizophrenia, schizoaffective disorder, psychotic disorder induced by substances or drugs or personality disorders (e.g., paranoid personality disorder), among others (Garety et al., 1999). These delusional thoughts have traditionally been considered as false beliefs that are hard to modify, expressed with absolute conviction and not amenable to reason (Freeman et al., 2013). Therefore, in the study of paranoid thoughts, primary cognition must be considered, since irrational beliefs may be the result of a negative evaluation of situations, relationships with others and their interpretations (Freeman and Garety, 1999; Freeman et al., 2002; Morrison et al., 2015). Metacognition should also been taken into account, since delusional thinking is linked to second-order cognition (thoughts about one’s thoughts) (Moritz et al., 2016). That is, delusions are also related to the pathological confidence linked to incorrigibility, and to the collection of less information before making a decision or “jumping to conclusions” (Garety et al., 1999; Freeman et al., 2002; Moritz and Woodward, 2005). This confidence in thoughts is especially important, given that, regardless of the content of what we think, trust (or doubt) guides the use of this content (Self-Validation Theory) (Briñol and Petty, 2003).

Since pathological confidence in one’s thoughts is a key mechanism of delusional thinking, many of the current therapies focus on trying to reduce it. One of the most recent therapies is developed by Moritz and Woodward (2007) and Moritz et al. (2013), called MyMCT (previously MCT). This therapy is based on making patients aware of the cognitive distortions linked to delusional thinking (e.g., “jump to conclusions” or “overconfidence” in errors). Subsequently, they are taught alternative coping strategies and information processing. Therefore, one of the objectives of this therapy is to reduce confidence in pathological thoughts, in order to alter delusional beliefs (Moritz et al., 2014a; Eichner and Berna, 2016). However, not only metacognitive therapies influence the processes of secondary cognition linked to paranoid thinking. From a cognitive point of view, the induction of doubt is one of the central cognitive effects of antipsychotics (e.g., Haloperidol) (Freeman et al., 2002). The antipsychotics also cause patients to detach from their thoughts, which allow them to treat thoughts as objects and diminish confidence in them (Kapur et al., 2005).

Therefore, generating doubt to reduce pathological confidence seems to be a good option in the treatment of chronic paranoid thinking. This reduction in confidence can occur through affective or cognitive (in)validation. Affective validation occurs when people use their thoughts because they feel good about them or like them (e.g., Briñol et al., 2007; Huntsinger et al., 2014). Therefore, dampening positive emotions reduces thought reliance. Cognitive validation takes place when people use their thoughts because they believe they are valid or correct (see Briñol et al., 2018 for a description of both types of validation).

The fundamental problem is that the doubt generated (both by metacognitive therapies and by antipsychotics) could not only be limited to delusional thoughts (reducing pathological confidence), it could also be extended to cognition in general (generating an extreme doubt). This would be especially relevant given that trust in thoughts is a fundamental variable for people’s positive psychological functioning and well-being (Taylor and Brown, 1988). In addition, paranoid thinking greatly affects patients’ quality of life (Karow and Pajonk, 2006).

For these reasons, studying the well-being of people who experience chronic paranoid thoughts is fundamental, regardless of their psychopathological evaluation (Chan et al., 2018). In fact, the presence of health not only requires absence of disease, but also having a state of complete physical, mental and social well-being (World Health Organization, 1946). To evaluate this state Corey Keyes developed the Complete State Model of Health (CSMH) (Keyes, 2005). According to this model, health is not a state characterized solely by the absence of disease, but also by the presence of indicators of hedonic well-being (hedonia) and eudaimonic well-being (positive functioning). The application of this model for the evaluation of patients with paranoid thinking has interesting consequences given that the CSMH defends that health and disease are not two poles of a single dimension, but two unipolar dimensions correlated with each other (two continua model of mental health). Therefore, the presence of pathology does not imply the absence of positive health. Although the CSMH has been widely applied and tested in different populations (Keyes, 2006, 2009; Bariola et al., 2017; de Vos et al., 2018), the existence of two different axes (positive mental health and mental illness) should be examined for each disorder or psychopathological process (e.g., McGaffin et al., 2015; Díaz et al., 2018) since there are cases where the existence of two axes is not met (e.g., Díaz et al., 2007).

Although it is especially interesting to apply the CSMH in the study of disorders related to paranoid thinking, to the best of our knowledge there are no studies that have proven the existence of two axes in these disorders. There is just one study in which the CSMH has been applied in the study of schizophrenia spectrum disorders (Chan et al., 2018), but in that study the existence of two-dimensions related to mental illness and positive mental health was not examined. For these reasons, the first objective of the present research is to apply the CSMH to a sample of patients with paranoid thinking to explore the two different unipolar dimensions: paranoid thinking and positive mental health (i.e., well-being indicators). In addition, we expect that paranoid thinking and positive mental health will be correlated.

Finally, our second objective is to study how metacognitive and pharmacological therapies influence confidence in thoughts of people with paranoid thinking. Based on previous research (Moritz et al., 2014a; Eichner and Berna, 2016) our next hypothesis is that therapies will reduce pathological confidence. However, we also expect that these therapies will generate extreme doubt in all thoughts, and not only the ones associated with delirium. Given that confidence is a key element of positive functioning, this adverse metacognitive effect of generalization of doubt would affect patients’ positive health (i.e., well-being). As a consequence, we expect doubt to partially mediate the effect of paranoid thinking on patients’ well-being.

## Materials and Methods

### Participants

Sixty participants (25 females and 35 males) with SCID-5 confirmed DSM-5 diagnosis related with paranoid thinking and without mood symptoms (i.e., Schizophrenia, Brief Psychotic Disorder, Delusional Disorder or Substance/Medication-Induced Psychotic Disorder) were recruited in the HGUCR. Recruitment was carried out between September 2016 and August 2017. Patients were hospitalized and were in a post-acute or stable phase of their disorders. Patients with a comorbid diagnosis of other mental disorders at the time the study was conducted were excluded (e.g., Major Depressive Disorder). In addition, participants were excluded if they could not complete questionnaires written in Spanish. Participants were recruited via letter of invitation explaining the project and the voluntary nature of participation. They were 25 women (42%) and 35 men (58%) between 18 and 76 years old. The mean age was 41.12 years (SD = 14.78). The maximum educational level reached to 57% of primary education, 20% higher no university education, 20% hold a university degree and 3% a PhD. Twenty patients had Schizophrenia, twenty Brief Psychotic Disorder, fifteen Delusional Disorder, and five Substance/Medication-Induced Psychotic Disorder. All participants were already prescribed atypical antipsychotic medication at baseline. Participants received a metacognitive therapy focused in overconfidence based on Well’s metacognitive therapy, which assumes that the cause of disorder is located at the metacognitive level not at the level of cognitive content (Wells and Matthews, 1996). The adaptation of the Wells’ metacognitive model of GAD (Wells, 1995) proposed by Morrison et al. (Morrison et al., 2014) was used. The exact duration of sessions varied, as a flexible use of the manuals is advocated, based on the individual case formulation (e.g., Normann et al., 2014; see Hutton et al., 2014, for a complete description of the therapy). On average, patients received 562 min of metacognitive therapy (SD = 51 min).

### Procedure

The study was approved by the ethics committee of the “Universidad de Castilla – La Mancha” (UCLM) and the HGUCR (Comité Ético de Investigación Clínica HGUCR-UCLM). All participants were informed that all collected information was confidential and anonymous, and signed an informed consent. To avoid possible order effects of the two sets of scales, half of the participants first completed the PIQ, and the PADS, and then the Satisfaction with Life Scale, the Positive Affect Scale and the Psychological Well-being Scales. The other half first completed the well-being scales and, then, proceeded to complete the PIQ and PADS.

### Measures

#### Paranoid Thinking

Participants filled the Spanish version (Fonseca-Pedrero et al., 2009) of the PIQ (McKay et al., 2006), a 10-item tool that was constructed according to comprehensive definitional considerations and criteria set out by Freeman and Garety (2000). The original PIQ and the Spanish version of the questionnaire have previously used with clinical and non-clinical samples, showing in both cases excellent reliability and validity as a brief measurement of paranoid thinking (i.e., persecutory ideation; Fonseca-Pedrero et al., 2009). Participants answered using a 5-point Likert scale response format ranging from 0 (very untrue) to 4 (very true). In addition, the Spanish version (Valiente et al., 2011) of the PADS (Melo et al., 2009); was used. This scale is a brief measure to assess both the severity of paranoid thinking (PADS-P) and the perceived deservedness of persecution (PADS-D) and is suitable for both clinical and non-clinical populations. The two subscales possess good internal consistency and factorial validity (e.g., Valiente et al., 2011). Participants were asked to rate each item on a 5-point scale ranging from 0 (certainly false) to 4 (certainly true).

#### Positive Health

According to the CSMH, to measure hedonia indicators, participants responded to the Satisfaction With Life Scale (Diener et al., 1985) and Positive Affect Scale. The Satisfaction With Life Scale (Spanish version; Cabañero et al., 2004) includes five items with adequate psychometric properties (e.g., Rodríguez-Carvajal et al., 2010). Participants responded to the items using a Likert scale ranging from 1 (strongly disagree) to 5 (strongly agree). Moreover, they completed the Positive Affect Scale (Spanish version; Díaz et al., 2007) indicating how frequently, during the last month, they have felt: calm and peaceful, extremely happy, in good spirits, cheerful, satisfied, and full of life. Participants answered the items using a response format ranging from 1 (never) to 5 (all the time). We also measured positive function indicators using the Psychological Well-being Scales (Ryff, 1989; Díaz et al., 2006). This instrument includes six dimensions of positive functioning (i.e., autonomy, self-acceptance, positive relations, environmental mastery, purpose in life and personal growth). Participants responded using a 6-point Likert scale anchored with 1 = strongly disagree and 6 = strongly agree. The structure of six dimensions (with or without a general factor) has been demonstrated through confirmatory factor analysis in previous research (Díaz et al., 2006, 2015; Van Dierendonck et al., 2008). Following Keyes (2005) and Díaz et al. (2018), to obtain a categorical measure of the presence (flourishing) or absence (languishing) of positive mental health, we considered the presence of each indicator when participant’s score in the scale was equal to or greater than the mean of the general Spanish population according to an aggregate data set composed by three samples (Blanco and Díaz, 2005; Díaz et al., 2006, 2007). The cut-off points for the categorical mental health indicators were: satisfaction with life = 3.28; positive affect = 3.36; autonomy = 4.24; self-acceptance = 4.31; positive relations = 4.58; environmental mastery = 4.32, purpose in life = 4.48; personal growth = 4.56. The presence of positive mental health required both criteria of hedonia and positive functioning. Moreover, each patient took part in a semi-structured interview based on the indicators proposed by Keyes (2005). The professionals who interviewed the patients codified the presence or absence of each indicator. Next, the presence or absence of positive health was tested based on the same criteria (CSMH categorical diagnosis; see Supplementary Table S1). The evaluations of well-being scales and semi-structured interviews converged for 58 patients (96.7%). In the two divergences found, we used the result of the semi-structured interview.

#### Doubt in General Thoughts

To assess participants’ subjective feeling of doubt in general thoughts two items anchored at not at all (1) and extremely (9) asking how much doubt they had in their general thoughts and how invalid they considered their general thoughts, were administered. Ratings were highly intercorrelated (*r* = 0.87), so they were averaged to create a composite doubt index. Higher values on this index indicated more doubt in general thoughts.

### Data Analysis

To test the two continua model of mental health (two different dimensions emerge from the measures used to assess positive mental health and paranoid thinking), we conducted an EFA. A critical methodological decision concerning EFA is the number of factors to retain. According to previous literature, we employed PA, which is one of the most accurate factor retention methods (e.g., Glorfeld, 1995; Hayton et al., 2004). Specifically, we calculated the mean and the 95th percentile for each of the eigenvalues of 100 randomly generated data sets using a SPSS syntax (O’connor, 2000). Next, we compared these random data eigenvalues to real data eigenvalues obtained from a PCA. In the PCA we extracted a number of factors equal to the number of scales entered into the analysis. Finally, we conducted an EFA based on the criterion established by the PA regarding the number of factors to be extracted. We employed *principal axis* as factor extraction method (Fabrigar et al., 1999) and *direct oblimin* as rotation method, since the emerged dimensions were expected to be correlated. We computed factor scores following the procedure proposed by Grice (2001). To study the relationship between paranoid thinking and well-being indicators, we used Pearson’s correlations, a contingency table, and a Fisher’s exact test. Finally, to test our hypothesis regarding the mediating role of doubt between paranoid thinking and patients wellbeing, we conducted a biased corrected bootstrapping procedure with 10,000 bootstrap re-samples using Hayes PROCESS macro (model 4; see Figure 2). PROCESS is a computational procedure for SPSS and SAS that implements moderation or mediation analysis as well as their combination in an integrated conditional process model (Shrout and Bolger, 2002; Preacher and Hayes, 2004; Hayes, 2013; Bajo et al., 2018). Some factors can produce spurious associations, particularly in a non-experimental study such as the present one. Therefore, demographic data, including sex, age, and education level, and DSM-5 diagnosis were introduced as covariates in mediation analysis.

## Results

**FIGURE 1:**
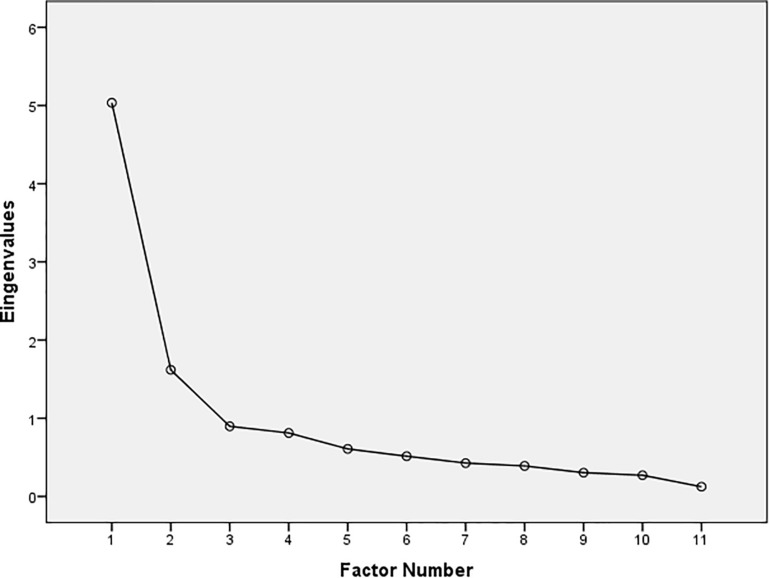
Scree plot.

Descriptive statistics (means and standard deviations) and Cronbach alpha coefficients (α) of paranoid thinking, well-being scales and doubt index are displayed in Table 1. As we can see in Table 2, the results of PA indicate that only the first and second eigenvalues of the real dataset exceeded random values. Scree Plot (Figure 1) confirms these results. Based on these findings, an EFA was conducted. As shown in Table 3, all the well-being scales essentially loaded on the first factor, explaining 46% of variance. On the other hand, all paranoid thinking scales loaded on the second factor, explaining 14% of the variance. These results support the two continua model of mental health. That is, mental health has two different unipolar dimensions: Positive mental health (i.e., Satisfaction With Life Scale, Positive Affect Scale and Psychological Well-being Scales; factor 1) and paranoid thinking (i.e., PIQ, PADS-P and PADS-D; factor 2). Correlation between the two factors was -0.46, which is an indicator of the strong existing relationship between paranoid thinking and positive health. To explore in more detail this relationship, we calculated the Pearson’s correlations of well-being scales and paranoid thinking scales (Table 4). The psychological well-being scales were all significantly correlated with PIQ, PADS-P and PADS-D (with the exception of the correlation between Personal Growth and PIQ). Precisely, the autonomy scale showed stronger relation to paranoid thinking than the rest of the scales. However, the subjective well-being scales showed a weak relationship with paranoid thinking. Only the relationship between life satisfaction and PIQ was significant.

**TABLE 1:** Means (M), standard deviations (SD), and Cronbach alpha coefficients (α) of paranoid thinking, well-being measures, and doubt index.

	***M***	***SD***	**α**
*Paranoid thinking*			
PIQ	7.97	8.32	0.94
PADS-P	1.68	0.97	0.87
PADS-D	0.91	0.75	0.87
*Subjective well-being*	3.21	0.64	0.75
Life satisfaction	3.06	0.95	0.83
Positive affect	3.31	0.75	0.74
*Psychological well-being*	4.18	0.77	0.89
Autonomy	4.03	0.90	0.70
Self-acceptance	4.21	1.11	0.69
Positive relations	3.83	1.14	0.71
Enviromental mastery	4.15	0.90	0.68
Purpose in life	4.33	0.99	0.74
Personal growth	4.62	0.86	0.71
Doubt index	5.23	2.37	0.93
			

**TABLE 2:** Parallel analysis.

**Eigenvalues**	**Random means**	**Real data**
1	1.77	5.04
2	1.52	1.62
3	1.35	0.90
4	1.20	0.81
5	1.06	0.61
6	0.95	0.52

**TABLE 3:** Exploratory factor analysis of paranoid thinking scales and well-being measures.

	**1**	**2**
PIQ		0.69
PADS-P	−0.44	0.91
PADS-D	−0.43	0.75
Satisfaction	0.42	
Positive affect	0.49	
Autonomy	0.63	−0.65
Self-acceptance	0.92	−0.44
Positive relations	0.50	−0.46
Enviromental mastery	0.73	−0.45
Purpose in life	0.75	
Personal growth	0.85	−0.41
Factors correlation		−0.46
% Variance	46	60

*Presented is the structure matrix of a principal axis extraction with *direct oblimin* rotation. The table only presents loadings above 0.40. N:*p* ratio = 5.5. All communalities >0.50 with the exception of PIQ (0.45), Satisfaction (0.30), Positive affect (0.35), and Positive relations (0.35).*

**TABLE 4:** Pearson’s correlations and 95% confidence intervals of paranoid thinking scales and well-being measures.

	**PIQ**	**PADS-P**	**PADS-D**
Subjective well-being	−0.29^∗^[-0.58-0.05]	−0.25[-0.500.03]	−0.20[-0.42-0.04]
Life satisfaction	−0.27^∗^[-0.48-0.03]	−0.13[-0.410.19]	−0.12[-0.380.15]
Positive affect	−0.18[-0.530.20]	−0.26[-0.520.03]	−0.25[-0.520.08]
Psychological well-being	−0.42^∗∗^[-0.60−0.21]	−0.56^∗∗^[-0.71-0.38]	−0.58^∗∗^[-0.72-0.44]
Autonomy	−0.47^∗∗^[-0.66-0.24]	−0.57^∗∗^[-0.71-0.40]	−0.54^∗∗^[-0.69−0.40]
Self-acceptance	−0.41^∗∗^[-0.63-0.19]	−0.51^∗∗^[-0.67-0.31]	−0.44^∗∗^[-0.61-0.27]
Positive relations	−0.30^∗^[-0.54-0.07]	−0.40^∗∗^[-0.60-0.16]	−0.44^∗∗^[-0.63-0.23]
Enviromental mastery	−0.30^∗^[-0.51-0.11]	−0.45^∗∗^[-0.65-0.23]	−0.50^∗∗^[-0.67-0.28]
Purpose in life	−0.30^∗^[-0.53-0.05]	−0.39^∗^[-0.60-0.12]	−0.46^∗∗^[-0.64-0.24]
Personal growth	−0.17[-0.480.11]	−0.32^∗^[-0.54-0.05]	−0.38^∗∗^[-0.57-0.18]

*^∗^*p* < 0.05; ^∗∗^*p* < 0.01.*

Having the existence of the two-dimensionality hypothesis confirmed, we expected that some patients with paranoid thinking could be assessed as healthy by meeting the criteria for the presence of positive health (categorical diagnostic approach). The categorical diagnosis requires the criteria of hedonia (a high level on positive affect and/or informed life satisfaction) and positive function (a high level on three or more of the psychological well-being indicators) to be met. To test this idea, we computed a contingency table of positive mental health (presence-absence) X disorder (Schizophrenia, Brief Psychotic Disorder, Delusional Disorder and Substance/Medication-Induced Psychotic Disorder) (Table 5). Notably, 19 patients (31.7%) were mentally healthy.

**TABLE 5:** Contingency table of mental disorder diagnosis and positive health.

		**Disorder**				
		**Schizophrenia**	**Delusional**	**Brief Psychotic**	**Substance**	**Total**
Positive health	Presence	6	5	6	2	19
	Absence	14	10	14	3	41
Total		20	15	20	5	

*Pearson’s χ^2^ = 0.23, *p* = 0.97. Fisher’s exact test *p* = 0.97.*

**FIGURE 2:**
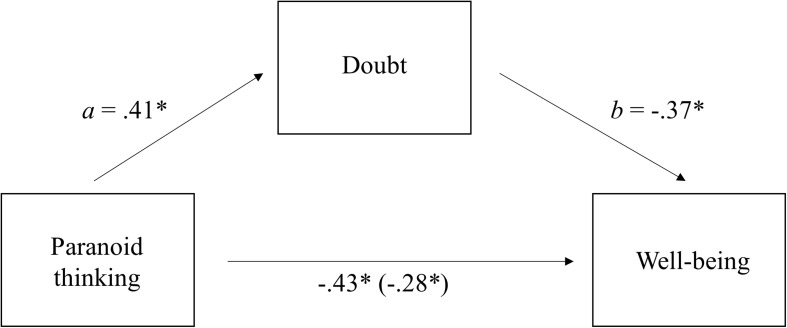
Doubt in one’s thoughts as a mediator between Paranoid Thinking and Well-being. Figure in the parenthesis (i.e., –0.28) is the direct effect of Paranoid Thinking on Well-being while accounting for the effect through the indirect path (^∗^*p* < 0.05).

Finally, applying the two continua model of mental health and according to our hypothesis (cognitive and pharmacological therapies reduce pathological confidence in all kind of thoughts, not only in pathological ones), we predicted that: (1) more paranoid thinking implies more external doubt (induced by longer metacognitive therapy sessions or more antipsychotics doses) in general thoughts; (2) more paranoid thinking implies less well-being; (3) more doubt implies less well-being; (4) doubt partially mediates the relationship between paranoid thinking and well-being. First, to check that metacognitive therapy was related to participants’ doubt in their own thoughts we correlated the total duration of therapy with the doubt index. Pearson correlation was significant (*r* = 0.58, *p* < 0.01), therefore longer duration implied greater doubt. Next, to test our hypothesis we conducted a biased corrected bootstrapping procedure using Hayes PROCESS macro (model 4) (Shrout and Bolger, 2002; Preacher and Hayes, 2004). This approach includes procedures that compute a 95% CI around the indirect effect and mediation is supported if this CI does not include zero. Paranoid thinking was the independent variable, well-being was the dependent variable, and doubt was the mediating variable (see Figure 2). As predicted, the data revealed that the 95% CI of the indirect effect (i.e., the path through the mediator) did not include zero (Indirect Effect a x b = 0.15, CI 95% = from -0.34 to -0.05), thus mediation by doubt is supported (Bajo et al., 2018).

## Discussion

Primary social cognition (e.g., the content or direction of thoughts) has been widely studied in paranoid thinking. In fact, there are different theories that have tried to explain the importance of social cognition in the origin and maintenance of this type of thinking (Penn et al., 1997; Freeman et al., 2013). However, the study of secondary cognition or metacognition has received less attention, although research in this concept has grown exponentially in the last decade. The interest in the study of metacognitive processes linked to paranoid thinking is basically related to pathological confidence that characterizes this type of thinking. This confidence is essential to understand paranoid delusions given that, regardless of the content of what we think, trust (or doubt) directs the use we make of this content (Self-Validation Theory) (Briñol and Petty, 2003). Therefore, increased confidence in a thought leads to more use of this though. In addition, pathological confidence causes thoughts to be rigid, and makes it very difficult to modify them (e.g., through the use of cognitive therapies, CBT) (Moritz et al., 2014a). As a result, different metacognitive interventions have been developed in recent years (e.g., Moritz et al., 2014a; Morrison et al., 2014). Despite the controversy over whether these therapies act at a cognitive or metacognitive level (e.g., Andreou et al., 2018; Capobianco and Wells, 2018), they focus on the generation of doubt in thoughts related to paranoid delirium (Moritz and Lysaker, 2018). Moreover, the induction of doubt is also one of the effects of antipsychotic drugs. For this reason, the main objective of this research was to analyze the effect of doubt (vs. confidence) caused by this type of therapies (i.e., metacognitive and pharmacological based on antipsychotics) on patients’ well-being and quality of life.

In order to analyze patients’ well-being, we applied the CSMH to the study of paranoid thinking. First, and according to our hypothesis, we verified one of the main axioms of the CSMH, the existence of two different axes: mental illness (paranoid thinking) and positive mental health (well-being indicators). Both Horn PA and EFA indicated the presence of two oblique factors. Although previous research applied CSMH in the study of schizophrenia spectrum disorders (Taylor and Brown, 1988), the existence of two-dimensions related to mental illness and positive mental health has not been proven. To the best of our knowledge, this is the first time that the existence of this axiom is confirmed. Despite this bi-dimensionality (mental illness – positive mental health), both factors were highly correlated. In this sense, the strongest relations were between psychological well-being and paranoid thinking (PADSP, PADSD, and PIQ). In particular, autonomy showed the greatest association with paranoid thinking. Furthermore, this indicator of psychological well-being showed a high factorial load (>0.60) in factor 2 (Paranoid Thinking). These results are consistent with previous research that indicates the strong impact of schizophrenia spectrum disorders on patients’ autonomy and quality of life (Prouteau et al., 2005; Carrión et al., 2013).

The existence of two different axes (mental illness – positive mental health) has interesting consequences. The first is related to the idea that the absence (or decrease) of paranoid thinking is not equivalent to the presence of health. Therefore, it is important to continue working to improve well-being and quality of life of patients with paranoid thinking, independently of the reduction of symptoms (Collins et al., 1991; Norman et al., 2000). From another perspective, a second consequence is that the presence of paranoid thinking does not necessarily imply the absence of positive health (i.e., subjective well-being and psychological well-being). In fact, according to our results, 19 out of 60 patients showed presence of positive health (flourishing) despite being diagnosed with disorders related to paranoid thinking. Therefore, patients with clinical symptoms of paranoid thinking may inform satisfactory levels of well-being and quality of life. A possible explanation of the high number of patients with presence of positive mental health is related to our sample, given that all patients were in a post-acute or stable phase of the disorders. Even so, other research has also found similar results (Chan et al., 2018).

Once the main axiom of the CSMH was verified, our main purpose was to evaluate the impact that metacognitive and pharmacological therapies based on antipsychotics have on patients’ well-being. Our results suggest that when patients received metacognitive and pharmacological treatment, more paranoid thinking implied more doubt in general thoughts. This relationship probably appeared because patients with more paranoid thinking required longer sessions of metacognitive therapy and higher doses of prescribed antipsychotic. These results seem to suggest that although the main metacognitive therapies focus on reducing pathological confidence, they could be affecting thoughts regardless of whether they were related to delirium or not. The same might happen with antipsychotics, that is, their effect on generating doubt may not be specific to pathological content. Consequently, the generalization of doubt in all thoughts could negatively affect patients’ well-being. This result should not be surprising because confidence in thoughts is a fundamental variable of positive functioning (Huppert and So, 2013). Indeed, doubt that patients reported in their thoughts (generated by the metacognitive and pharmacological treatment) partially mediated the effect of paranoid thinking on well-being. Therefore, despite the efficacy shown by antipsychotics (first or second generation; Leucht et al., 2009) and metacognitive therapies (Moritz et al., 2014a), they can produce a generalization of doubt which in turn can negatively affect patients’ well-being and quality of life, at least in the beginning of the treatment. Previous research (Moritz et al., 2014b) indicated that in the long term there could be a “sleeper” effect of metacognitive therapy that would increase patients’ self-esteem and quality of life. Future studies could explore if this “sleeper” effect is related to the recovery of confidence in thoughts not related to delirium. In this sense, to improve patients’ well-being and quality of life, it seems interesting to design interventions to increase confidence in thoughts not related to delirium.

Despite the contribution described above, the present work has several limitations. The first one is related to the sample size. We determined the sample size taking into account the difficulty to access hospitalized patients with paranoid thinking who would agree to collaborate and fill out the informed consent. In fact, most studies related to paranoid thinking and schizophrenia tend to have relatively small samples (Kay et al., 1987; Briggs et al., 2008; Dias et al., 2013; Eisenacher and Zink, 2017). Although sample size may have affected some of our analyses, it did not compromise the obtained conclusions. Regarding the factorial analyses carried out, although the ratio N:p is relatively low (5.5), the communalities are greater than 0.50 (with the exceptions of PIQ = 0.45, Satisfaction = 0.30, Positive Affection = 0.35, and Positive Relationships = 0.35). These results indicate an acceptable factor recovery (MacCallum et al., 1999; Hogarty et al., 2005). In addition, with respect to external validity, different studies have found similar results showing the emergence of two different but related factors of positive health measures (i.e., well-being) and pathology (Keyes, 2005; Bajo et al., 2018). Concerning the contingency table between diagnosis of mental disorders and positive health, we used a Fisher’s exact test precisely because we expected low values in the cells of the contingency table. Regarding the correlation analyses, we have included CIs to measure the impact of the sample size on the results (Altman and Gardner, 1988). Finally, in relation to the mediation analysis, the smaller the sample size, the lower are the probabilities of finding a statistically significant mediation (Shrout and Bolger, 2002). Since our research reveal the expected mediation effect, this suggests that it is unlikely our study lacked sufficient power to support the null hypothesis. Another limitation of our study is that we did not use social well-being measures to evaluate positive mental health. Given the special characteristics of our sample, we decided not to evaluate this aspect, since we did not want cognitive fatigue to affect the results. Lastly, a final limitation of our study is that patients received simultaneously pharmacological treatment and metacognitive therapy. As a consequence, we could not study the effect of the generalization of doubt in isolation for each treatment. Probably, the effect of generalization of doubt is produced in both treatments in an isolated way since the cognitive effect of the antipsychotics’ confidence reduction does not seem to be specific for the paranoid thoughts, and there is a high probability that metacognitive therapy also affects thoughts unrelated to delusions (Moritz et al., 2014a, 2016). In fact, we found a significant relationship between the duration of metacognitive treatment and doubt in general thoughts. Future investigations should further explore this question.

## Conclusion

In this study we verified, for the first time, one of the main axioms of the CSMH: positive mental health and paranoid thinking are not two poles of a single dimension, but two unipolar dimensions correlated with each other. Therefore, the absence (or reduction) of paranoid thinking does not imply the presence of health. As a consequence, it is important to continue working to improve the well-being and quality of life of patients with paranoid thinking, independently of the reduction of symptoms. In addition, the presence of paranoid thinking does not necessarily imply the absence of positive health. That is, people with paranoid thinking can experience satisfactory levels of well-being. Once the main axiom of CSMH was verified, our main purpose was to evaluate the impact that metacognitive and pharmacological therapies based on antipsychotics have on patients’ well-being. Despite the efficacy shown by metacognitive therapies (Moritz et al., 2014a) and antipsychotics (first or second generation; Leucht et al., 2009), it seems that they not only reduce pathological confidence, but can also affect other thoughts not linked to delirium. This effect of generalization of doubt in all thoughts must be controlled, given that it negatively affects patient’s well-being and quality of life.

## Data Availability

The datasets used and/or analyzed during the current study are available from the corresponding author on reasonable request.

## Ethics Statement

This study was part of a research project funded by the Spanish Ministry of Education and Science, and was approved by the ethics committee of the “Universidad de Castilla – La Mancha” (UCLM) and the HGUCR (“Comité Ético de Investigación Clínica, HGUCR – UCLM”). All participants completed an informed consent form, assuring them that all information they provided would remain confidential and anonymous.

## Author Contributions

DD and LA-A conceived the study design. LA-A collected the data. DD, LA-A, and LB-F drafted the manuscript. MB, MS, and LA-A performed the data analysis. MS, MB, and TR-C contributed to the critical revisions of the manuscript. All authors discussed the results, implications, and literature, and approved the final version of the manuscript for submission.

## Conflict of Interest Statement

The authors declare that the research was conducted in the absence of any commercial or financial relationships that could be construed as a potential conflict of interest.

## Abbreviations

CBT: cognitive-behavioral therapy
CI: confidence interval
CSMH: Complete State Model of Health
DSM: Diagnostic and Statistical Manual of Mental Disorders
EFA: exploratory factor analysis
HGUCR: Hospital General Universitario de Ciudad Real
GAD: generalized anxiety disorder
MCT and MyMCT: metacognitive training
PA: parallel analysis
PADS: Persecution and Deservedness Scale
PCA: principal component analysis
PIQ: Persecutory Ideation Questionnaire
SCID-5: Structured Clinical Interview for DSM-5.
