# Supplementary material for: Paranoid Thinking and Wellbeing. The Role of Doubt in Pharmacological and Metacognitive Therapies
Source: Front Psychol. 2019 Sep 12;10:2099. doi: 10.3389/fpsyg.2019.02099 (PMC6751329; doi:10.3389/fpsyg.2019.02099)
Supplement: Supplementary file 1 [file Table_1.pdf]

Categorical Diagnosis of Mental Health (adapted from Keyes, 2005)

Diagnostic criteria		Symptom description
Hedonia: requires high level on at least one symptom scale (Symptoms 1 or 2)	1	Regularly cheerful, in good spirits, happy, calm and peaceful, satisfied, and full of life (positive affect past 30 days).<br>*Examples of questions of the semi-structured interview:*<br>How did you feel during the last month?<br>How easy is it for you to remember a positive event?
	2	Feels happy or satisfied with life overall or domains of life (avowed happiness or avowed life satisfaction).<br>*Examples of questions of the semi-structured interview:*<br>How satisfied are you with your life as a whole?<br>How many things of your life would you change?
Positive functioning: requires high level on three or more symptom scales (Symptoms 3–8)	3	Exhibits self-direction that is often guided by his or her own socially accepted and conventional internal standards and resists unsavory social pressures (autonomy).<br>*Examples of questions of the semi-structured interview:*<br>How easy is it for you to maintain your opinion in discussions with other people?<br>To what extent do you think that other people's opinions make you change your mind?
	4	Holds positive attitudes toward oneself and past life and concedes and accepts varied aspects of self (self-acceptance).<br>*Examples of questions of the semi-structured interview:*<br>How positive is your attitude toward yourself?<br>What aspects of yourself would you change?
	5	Has warm, satisfying, trusting personal relationships and is capable of empathy and intimacy (positive relations).<br>*Examples of questions of the semi-structured interview:*<br>Do you have close friends you can trust?<br>If you have a romantic partner, how intimate is your relationship?
	6	Exhibits capability to manage complex environment, and can choose or manage and mold environments to suit needs (environmental mastery).<br>*Examples of questions of the semi-structured interview:*<br>Do you have a sense of control over your life?<br>Are you capable to search for new social environments or modify current ones in a way that allows you to achieve your goals?
	7	Holds goals and beliefs that affirm sense of direction in life and feels that life has a purpose and meaning (purpose in life).<br>*Examples of questions of the semi-structured interview:*<br>Do you feel the things you do in your life are worthwhile?<br>Could you list the goals you have in life?
	8	Shows insight into own potential, sense of development, and open to new and challenging experiences (personal growth)<br>*Examples of questions of the semi-structured interview:*<br>Do you think you grow as a person over time?<br>Do you think you have developed most of your skills?
